# The moderating role of knowledge sharing behavior between innovative behavior and nursing competency: a cross-sectional study

**DOI:** 10.3389/fpsyg.2026.1808847

**Published:** 2026-08-19

**Authors:** Lu Bian, Yuan Qian, Yujuan Lou, Mingxia Xia, Yun Zhou, Ying Zhang, Longhua Yuan, Dandan Xia

**Affiliations:** 1 Department of Science and Education, Gaochun Hospital Affiliated to Jiangsu University, Nanjing, Jiangsu, China; 2 Department of Nursing, Affiliated Hospital of Jiangnan University, Wuxi, Jiangsu, China; 3 Department of Nursing, Gaochun Hospital Affiliated to Jiangsu University, Nanjing, Jiangsu, China; 4 College of Nursing, Qilu Medical University, Zibo, Shandong, China

**Keywords:** clinical nurses, innovative behavior, knowledge sharing behavior, moderating role, nursing competency

## Abstract

**Background:**

The association between nurses’ innovative behaviors and nursing competence has been demonstrated. However, the role of knowledge-sharing practices has been overlooked. Therefore, the purpose of this study is to explore the moderating role of knowledge sharing behavior between innovative behavior and nursing competency among clinical nurses.

**Methods:**

In this cross-sectional study, 258 clinical nurses from three tertiary hospitals in Nanjing and Wuxi were selected using convenience sampling approach. The scales used included the Nurse Innovative Behavior Scale, Knowledge Sharing Behavior Scale, and Nursing Competence.

**Results:**

The scores for innovative behavior, nursing competency, and knowledge sharing behavior were (39.19 ± 5.98), (84.62 ± 10.61), and (41.91 ± 11.62), respectively. A positive correlation was observed between innovative behavior and nursing competency (*r* = 0.301, *p* < 0.05). The relationship between knowledge sharing behavior and both innovative behavior and nursing competency was positive (*r* = 0.268, *r* = 0.365, *p* < 0.01). The interaction between innovative behavior and nursing competency was positively moderated by knowledge sharing behavior (interaction term *β* = 0.219, *p* = 0.031). Innovative behavior showed a stronger positive association with nursing competency in settings with high levels of knowledge sharing (*β* = 0.448, *p* < 0.001).

**Conclusion:**

Knowledge sharing behavior positively moderates the predictive association between innovative behavior and nursing competency. A supportive knowledge-sharing system should be a focus for nursing managers to facilitate the effective integration of innovative behavior into nursing proficiency.

## 1 Introduction

Amidst the rapid advancements in medical technology and the ongoing refinement of nursing practice systems, clinical nurses are progressively evolving from traditional roles as skill executors to multifaceted professionals endowed with innovative thinking and specialized expertise (Wang et al., 2025). Homans’s Social Exchange Theory (SET) posits that employees engage in voluntary information exchange and resource sharing to obtain reciprocal professional support and growth rewards, which lays the theoretical foundation for nurses’ knowledge sharing interactions within clinical teams (Ahmad et al., 2022). Organizational Learning Theory (OLT), initially proposed by Argyris, further explains that individual innovative attempts cannot permanently improve professional ability alone; only through team-level knowledge integration, communication and reflection can scattered innovative experience be converted into stable comprehensive competency (Abdelwahab Ibrahim El-Sayed et al., 2024; Argyris and Schön, 1997). Innovative behavior in nursing, characterized by the proactive identification of clinical issues, exploration of improvement strategies, and promotion of practical optimization, has emerged as a critical catalyst for enhancing the quality of nursing care and advancing professional development (Zhu et al., 2025; Lu et al., 2025). Research suggests that the innovative behavior exhibited by nurses may be associated with improved quality of nursing services through the optimization of workflows and refinement of techniques. Furthermore, such behavior is positively correlated with stronger complex clinical decision-making capacity, which correlates with higher nursing competency levels (Ho et al., 2019; Liu et al., 2024). Nursing competency, characterized by the comprehensive integration of nurses’ knowledge, skills, attitudes, and values, is intrinsically linked to patient safety and health outcomes. It plays a pivotal role in advancing the development of the nursing workforce and the nursing profession as a whole.

Although prior literature has confirmed the direct positive link between nurses’ innovative behavior and nursing competency, most existing studies only focused on their linear correlation and ignored potential boundary conditions that may strengthen or weaken this relationship. According to Organizational Learning Theory, individual innovation may have limited long-term competency association in the absence of team knowledge circulation. Therefore, a critical unanswered question remains: under what organizational interactive conditions is innovative behavior more strongly associated with nursing competency? Knowledge sharing behavior, defined as the process through which individuals generate, apply, and disseminate professional information via interpersonal and organizational exchanges to amplify collective knowledge value (Guo et al., 2020; Abdelwahab Ibrahim El-Sayed et al., 2024), is a typical situational boundary variable that may moderate the innovation-competency association. Knowledge sharing behavior is crucial for the flow and integration of knowledge within organizations and may significantly moderate the transition from innovative behavior to competency. Through the process of knowledge sharing, innovative experiences can be disseminated, integrated, and reconstructed within teams, thereby not only expediting the clinical implementation of novel methodologies but also fostering continuous learning and reflection among nurses through interactive engagement. This may be associated with a stronger positive influence of innovative actions on building professional competence (Asurakkody and Hee, 2020). Consequently, examining the moderating role of knowledge-sharing behavior in the relationship between innovative behavior and nursing competency is of substantial theoretical and practical importance for developing a nursing management system that fosters innovation and encourages knowledge dissemination.

Three distinct research gaps can be summarized from existing evidence: First, most nursing studies only test the direct correlation between innovative behavior and nursing competency, lacking empirical exploration of boundary moderating factors that alter this association. Second, few domestic cross-hospital surveys combine Social Exchange Theory and Organizational Learning Theory to systematically interpret the role of knowledge sharing in the association between individual innovative practice and comprehensive nursing competency. Third, prior relevant research failed to distinguish the differentiated predictive intensity of innovative behavior under high versus low knowledge sharing environments, lacking quantitative evidence from multi-center tertiary hospital clinical nurses in Eastern China.

This study involved the selection of clinical nurses from three tertiary hospitals located in Nanjing and Wuxi as the subjects of the research. Utilizing a cross-sectional survey combined with structural equation modeling analysis, this study seeks to explore the associations between innovative behavior and nursing competency. Additionally, it examines the moderating role of knowledge-sharing behavior, thereby offering a theoretical foundation and practical insights for nursing management practices.

## 2 Methods

### 2.1 Design

A cross-sectional study was performed in June 2025 to analyze the associations between innovative behavior, knowledge sharing, and nursing competency in clinical nurses.

### 2.2 Participants

We utilized a convenience sampling approach to recruit clinical nurses from internal medicine, surgery, emergency, obstetrics and gynecology, pediatrics and other departments of three Grade III-A hospitals located in Nanjing and Wuxi, China. The inclusion criteria were as follows: (1) participants were required to be registered nurses possessing a valid nursing license; (2) participants must have provided informed consent; and (3) participants needed to have a minimum of 1 year of clinical work experience. The exclusion criteria comprised: (1) nurses who were interns, trainees, or on external study leave; and (2) head nurses or other managerial personnel. According to Kendall’s criterion, the sample size for multivariate analysis should be 5 to 10 times the number of variables (Kline, 2023). There were 17 predictor variables in this study. The basic sample demand without attrition ranged from 85 (17 × 5) to 170 (17 × 10). Considering a projected 20% invalid questionnaire rate, we divided the basic sample quantity by 0.8 to get the adjusted sample threshold of 107–213 participants. The valid sample of 258 collected in this research fully meets this standard.

### 2.3 Measurements

#### 2.3.1 General information questionnaire

A self-developed general information questionnaire was independently designed by the research team, referencing demographic measurement tools adopted in published domestic cross-sectional nursing studies. The questionnaire contains 6 items covering basic demographic and occupational characteristics: age, years of clinical work experience, professional title, highest educational attainment, participation in research projects, and clinical working department. All items were reviewed and revised by two senior nursing department managers to ensure rationality and clinical suitability of the setting options.

#### 2.3.2 Nurse innovative behavior scale

This 10-item scale was developed by Bao et al. (2012) exclusively for Chinese clinical nurses and has been widely validated across Chinese tertiary hospitals without cross-cultural foreign versions. No item adaptation was conducted in this study, and there are no reverse-coded items. It includes three dimensions: idea generation, seeking support, and idea implementation. Participants rated items on a 5-point Likert scale (1 = never to 5 = frequently). Total scores range 10–50, with higher scores representing stronger innovative behavior. The Cronbach’s *α* of the scale was 0.879.

#### 2.3.3 Knowledge sharing behavior scale

The knowledge sharing behavior scale that was initially developed by Yi has gained extensive use across organizational, academic and research environments. In Chen and Wu (2015) completed its Chinese translation and revision to fit the context of clinical nursing staff in China. The four-factor structure of the scale includes written contribution (3 items), organizational communication (7 items), personal interaction (4 items), and community practice (5 items). Participants respond to each item via a 5-point Likert scale anchored at 1 (never) and 5 (always), yielding aggregate scores from 19 to 95. The Cronbach’s *α* value of the entire scale was reported as 0.923.

#### 2.3.4 Nursing competency scale

This scale was localized by our team based on international nursing competency tools from Bae et al. (2023). It contains 23 items without reverse-scored items, covering five dimensions: professional skills, interpersonal relationships, managerial capability, educational guidance, and research competence. Each item adopts a 1–5 Likert scoring; subscale scores are the sum of corresponding items, and the total score equals the sum of all subscales, with higher scores indicating stronger competency. The scale demonstrated robust psychometric properties, evidenced by a Cronbach’s *α* of 0.908, a split-half reliability of 0.988, and a test–retest reliability of 0.967 (Wang et al., 2022).

### 2.4 Data collection

Once we received ethical approval, we used an online survey platform to distribute the questionnaire. Data quality control measures included mandatory items to avoid missing values and one response per IP restriction. Questionnaires finished within 10 min or containing straight-lining responses (uniform repetitive scoring) were eliminated. Data collection spanned June to October 2025. All valid questionnaires were manually checked for logical consistency before analysis. All questionnaires were filled out anonymously without collecting participants’ names, ward numbers or employee IDs. The anonymous design helped reduce social desirability bias and improved the authenticity of self-reported responses.

### 2.5 Statistical analysis

The data were processed with SPSS 26.0 and AMOS 26.0. Prior to formal hypothesis testing, Harman’s single-factor test was performed to examine common method bias. All items of the three scales were loaded into an exploratory factor analysis without rotation. The first extracted common factor below the critical threshold of 40%, indicating no severe common method bias in this study. Demographic and scale data were summarized using descriptive statistics, while Pearson correlation analysis was used to explore the relationships between key variables. To eliminate multicollinearity between the independent variable, moderator and interaction term, mean centering was conducted for innovative behavior and knowledge sharing before constructing their cross-product interaction term. Hierarchical multiple regression was adopted to verify the moderating effect. Confirmatory factor analysis was conducted via AMOS to assess convergent and discriminant validity. For the measurement model, standard SEM modification procedures were followed: model fit indices were checked iteratively, and theoretically reasonable error covariances were added based on modification indices to optimize overall model fit. The Moderating role of knowledge sharing behavior was tested through structural equation modeling (SEM) using maximum likelihood estimation. The assessment of model fit involved χ^2^/df, RMSEA, GFI, NFI, IFI, and CFI. Common SEM fit criteria were adopted for model evaluation: acceptable thresholds were χ^2^/df < 5, RMSEA < 0.08, GFI, NFI, IFI and CFI > 0.90 (Yuhuan et al., 2022). Further analysis of moderation and estimation of 95% confidence intervals were done using simple slope analysis and bootstrapping with 5,000 resamples. The significance level was set at *α* = 0.05.

## 3 Results

### 3.1 General characteristics of participants

A total of 280 questionnaires were handed out, and 258 valid responses were obtained, leading to an effective response rate of 92.14%. The participants’ demographic and professional details are displayed in Table 1.

**Table 1:** General characteristics of clinical nurses (*N* = 258).

Item	Category	*N*	Percentage (%)
Age (years)	≤25	26	10.08
	26–30	69	26.74
	31–35	114	44.18
	36–40	31	12.02
	41–45	18	6.98
Years of work	≤1	25	9.69
	2–5	24	9.30
	6–10	104	40.31
	11–15	105	40.70
Professional title	Nurse	20	7.75
	Senior Nurse	5	1.94
	Supervisor Nurse	177	68.60
	Deputy Chief Nurse or above	56	21.71
Highest level of education attained	Junior College	17	6.59
	Bachelor’s degree	217	84.11
	Master’s degree or above	24	9.30
Participation in the research project	No	180	69.77
	Yes	78	30.23

### 3.2 Descriptive statistics and correlations

The scores for innovative behavior, nursing competency, and knowledge sharing behavior are shown in Table 2. According to Pearson correlation analysis, there was a positive correlation between innovative behavior and both nursing competency (*r* = 0.301, *p* < 0.05) and knowledge sharing behavior (*r* = 0.268, *p* < 0.05). A positive relationship was observed between knowledge sharing behavior and nursing competency (*r* = 0.365, *p* < 0.01).

**Table 2:** Descriptive statistics of innovative behavior, nursing competency, and knowledge sharing behavior (*N* = 258).

Variable	Item	Item score (Mean ± SD)	Total score (Mean ± SD)
Nursing competency	Professional skills	21.89 ± 2.91	84.62 ± 10.61
	Interpersonal skills	17.63 ± 2.47	
	Management ability	17.28 ± 2.59	
	Educational guidance	13.03 ± 1.95	
	Research competence	14.79 ± 2.79	
Innovative behavior	Idea generation	16.27 ± 2.18	39.19 ± 5.98
	Seeking support	15.61 ± 2.79	
	Idea implementation	7.31 ± 1.82	
Knowledge sharing behavior	Written contribution	9.86 ± 3.63	41.91 ± 11.62
	Organizational communication	10.72 ± 3.43	
	Personal interaction	12.13 ± 2.85	
	Community practice	9.18 ± 3.80	

### 3.3 Moderating role of knowledge sharing behavior

A structural equation model was created with AMOS 26.0, featuring nursing competency as the outcome variable, innovative behavior as the predictor, and knowledge sharing behavior as the moderating factor. The interaction term consisted of the product of innovative behavior and knowledge sharing behavior. The model’s fit indices were impressive: χ^2^/df = 2.370 (<5.000), RMSEA = 0.073 (<0.080), GFI = 0.907, NFI = 0.923, IFI = 0.954, CFI = 0.954, all meeting the recommended acceptable thresholds above 0.90. These values indicated good overall model fit.

The analysis of the path revealed that innovative behavior was positively associated with nursing competency significantly (*β* = 0.229, *p* = 0.001). Nursing competency was significantly and positively associated with knowledge sharing behavior (*β* = 0.224, *p* < 0.001). Significantly, the interaction term exhibited a positive effect (*β* = 0.219, *p* = 0.031), which suggests a moderating role of knowledge sharing behavior. Table 3 provides detailed results.

**Table 3:** Structural equation model path analysis results.

Path relationship	Unstandardized coefficient	Standardized coefficient (*β*)	SE	C.R.	*p-*value
Nursing competency ← Innovative behavior	0.472	0.229	0.135	3.504	0.001
Nursing competency ← Knowledge sharing behavior	0.181	0.224	0.055	3.318	<0.001
Nursing competency ← Interaction term*	0.142	0.219	0.066	2.154	0.031

Interaction term = Innovative behavior × Knowledge sharing behavior. *indicates statistical significance at *p* < 0.05.

### 3.4 Simple slope analysis

**Figure 1:**
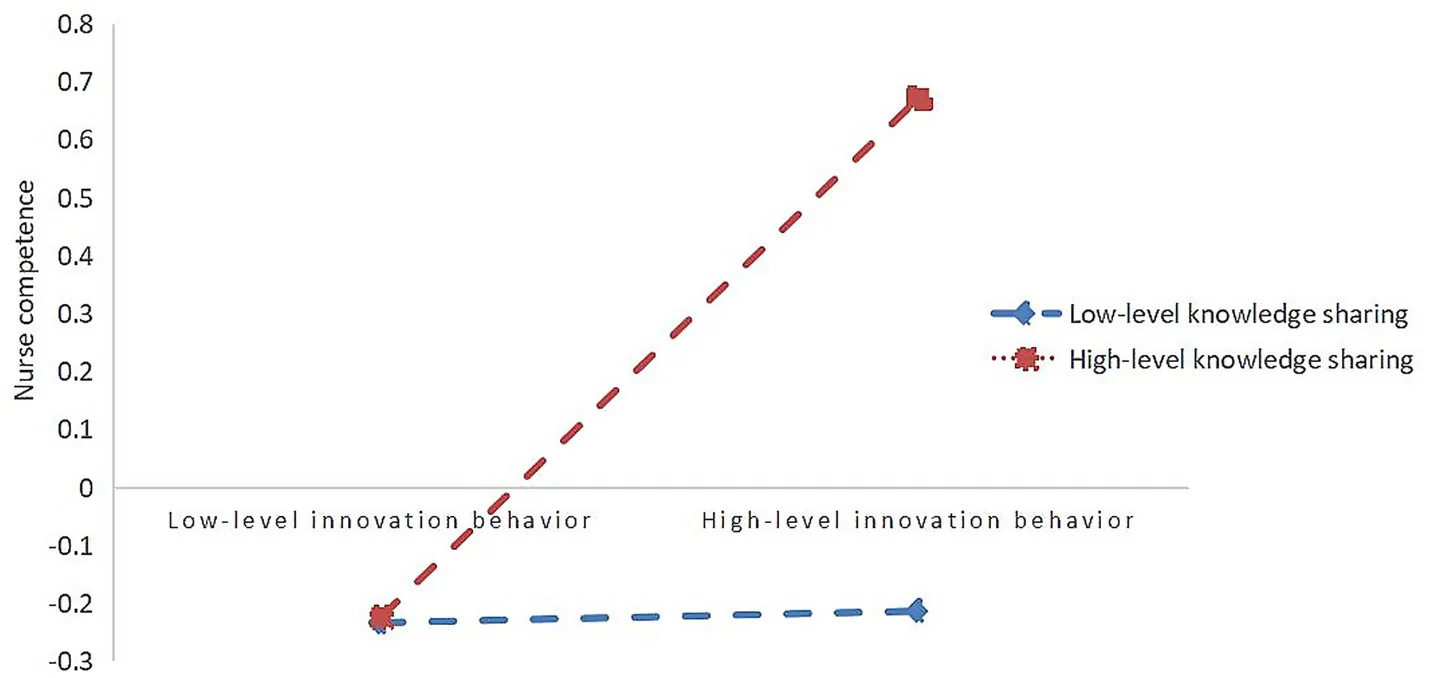
Simple slope plot of moderating effect.

**Figure 2:**
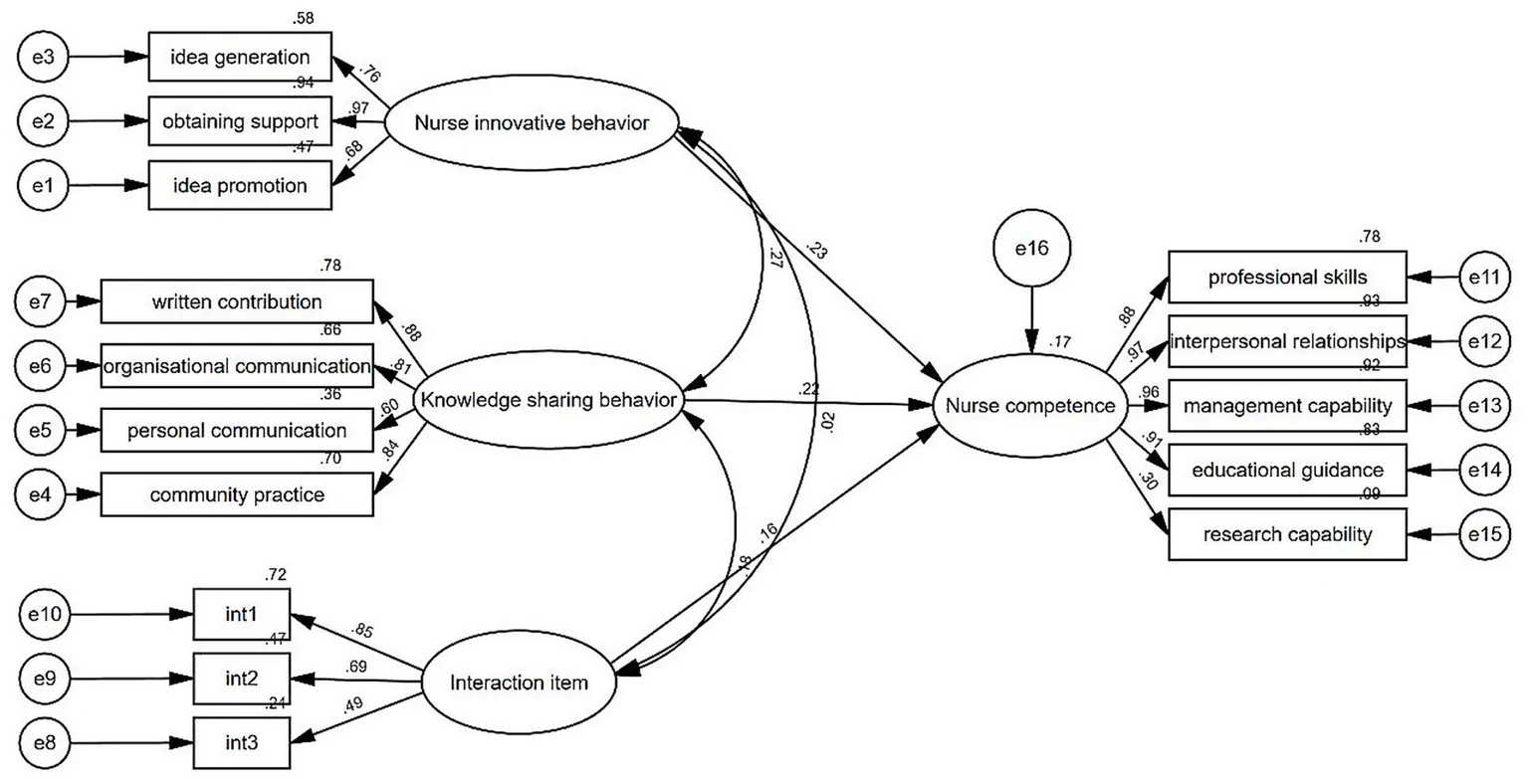
Schematic diagram of the moderating effect of knowledge sharing behavior between nurses’ innovative behavior and nursing competency.

In order to delve deeper into the Moderating role, we executed a simple slope analysis by dividing participants based on their knowledge sharing behavior scores (mean ± SD). Results demonstrated that with increased knowledge sharing, the positive association between innovative behavior and nursing competency was stronger (*β* = 0.448, *p* < 0.001). Conversely, when knowledge sharing was minimal, the effect was insignificant (*β* = 0.011, *p* = 0.909). This suggests that increased knowledge sharing is associated with a stronger positive association between innovative behavior and nursing skills. The results are summarized in Table 4 and Figures 1, 2.

**Table 4:** Simple slope analysis of the moderating effect.

Group	Effect size	95% CI		*p-*value
		Lower limitation	Upper limitation	
High knowledge sharing group	0.448	0.188	0.699	<0.001
Medium knowledge sharing group	0.229	0.104	0.347	<0.001
Low knowledge sharing group	0.011	−0.289	0.287	0.909

## 4 Discussion

### 4.1 Analysis of the current status of innovative behavior, nursing competency, and knowledge sharing behavior

According to the study, clinical nurses’ innovative behavior had a total score of (39.19 ± 5.98), reflecting a medium to high level. The dimension of idea generation received the highest score (16.27 ± 2.18), indicating nurses’ relatively high problem awareness and innovative thinking in clinical settings. The seeking support dimension score (15.61 ± 2.79) indicates that nurses are competent in obtaining resources and collaborative assistance. In comparison, the score for implementing ideas was low (7.31 ± 1.82), suggesting that nurses may face challenges in bringing innovative concepts to fruition. This might be associated with issues like high workloads, insufficient resources, and organizational barriers (Yan et al., 2022). This dimensional distribution pattern is highly consistent with the cross-sectional findings of Wang et al. (2024), who also reported that Chinese frontline nurses scored highest on idea generation yet lowest on idea implementation using the same Nurse Innovative Behavior Scale, and similarly attributed the implementation gap to heavy clinical workload and insufficient organizational innovation support mechanisms. Variations in innovative behavior scores were noted among different professional titles, with senior nurses and those in higher positions achieving higher scores, possibly due to their accumulated work experience and greater professional autonomy. This demographic difference aligns with the multicenter research of Li et al. (2025), whose latent profile analysis verified that nurses with senior professional titles exhibited significantly higher overall innovative behavior, as richer clinical experience and greater workplace autonomy create more opportunities for them to put forward and practice improvement plans. Amidst the rising importance of innovative literacy in nursing education and practice, clinical nurses have progressively developed an awareness of innovation and a readiness to explore (Atasoy et al., 2023).

Nursing competency had a total score of (84.62 ± 10.61). The dimension of professional skills scored the highest at (21.89 ± 2.91), showing that clinical nurses are proficient in operational skills, patient observation, and emergency situations. Nurses demonstrate strong competence in teamwork, coordination, and communication, as shown by their interpersonal skills score of (17.63 ± 2.47) and management ability score of (17.28 ± 2.59). With a score of (13.03 ± 1.95), the educational guidance dimension shows that clinical teaching abilities could be improved. The research competence dimension had a lower score of (14.79 ± 2.79), indicating that nurses have considerable room for improvement in research design, academic writing, and translating evidence. The reason might be an insufficiently developed nursing research training system combined with demanding clinical workloads. Flodén et al. (2026) conducted a cross-sectional study among 615 nurses at a Swedish regional teaching hospital using the Nurse Professional Competence Scale, reporting a total mean score of 84.3 (79.3–90.0)—strikingly close to the (84.62 ± 10.61) in the present study. Notably, their findings parallel the dimensional pattern observed here: the highest scores were in “Value-based nursing care” and “Medical and technical care, “while the lowest were in “Care pedagogics” and “Development, leadership and organization of nursing care”. This cross-cultural consistency suggests that the strength in clinical-technical skills and the relative weakness in educational guidance and leadership may be shared characteristics of nursing competence across different health systems. Regarding research competence, Liu et al. (2025) surveyed 436 pediatric clinical nurses in China using the Self-assessment Scale for Nursing Staff’s Scientific Research Ability, reporting a total score of 47.03 ± 13.66, which was characterized as “at a low level”. Their regression analysis further identified educational level, professional title, and the frequency of receiving scientific research training as independent influencing factors. These findings corroborate the present study’s observation that research competence is an area requiring substantial improvement, and they empirically support the suggested explanation that an underdeveloped research training system—particularly for nurses with lower educational backgrounds—contributes to this deficiency.

The total score for knowledge sharing behavior was (41.91 ± 11.62), with the personal interaction dimension scoring the highest at (12.13 ± 2.85), suggesting that clinical nurses prefer informal face-to-face exchanges and mentoring for transferring knowledge. In contrast, the scores of written contribution and community practice dimensions were relatively low, which reflected nurses’ low participation in structured and systematic forms of knowledge sharing, such as writing abstracts or participating in academic communities. This may be related to the high-intensity and fast-paced nature of nursing work, underdeveloped internal knowledge management mechanisms, and an incomplete sharing culture within organizations (Karanika-Murray et al., 2023). When discussing the universality of this finding, the present sample only covers frontline bedside nurses from tertiary hospitals in Jiangsu Province. This clear preference for casual interpersonal knowledge exchange and low engagement in formal written sharing may not hold equally for other nurse groups. For example, nurse educators, research nurses, and nurse managers with fewer routine bedside tasks have more spare time to compile clinical experience and join academic communities, which may narrow the gap between personal interaction and formal knowledge sharing dimensions. Meanwhile, nurses in primary care or community medical institutions face different work pressure and organizational support systems, so their structural patterns of knowledge sharing could differ substantially from the participants in this study. Therefore, encouraging the shift from personal communication to organized documentation in knowledge sharing is crucial for improving nurses’ knowledge management.

### 4.2 Correlations among innovative behavior, nursing competency, and knowledge sharing behavior

The Pearson correlation analysis results of this study showed that innovation behavior was significantly positively correlated with nursing competency (*r* = 0.301, *p* < 0.05). Based on Cohen’s criteria, *r* = 0.301 reflects a medium positive effect size, indicating a moderate association between the two variables, suggesting a positive predictive association between innovative behavior and comprehensive nursing competency, which was consistent with the results of elbus’s study (Elbus et al., 2024). Innovative behavior involves a continuous cycle of learning, reflecting, and enhancing everyday tasks. Through the circular process of “finding problems – proposing solutions – practical verification, “nurses may deepen their understanding of professional knowledge, expand the application scope of clinical skills, and enhance decision-making abilities in complex situations, which may contribute to multidimensional growth in competency (Gonzalez-Garcia et al., 2025). Although trial-and-error innovation may bring temporary work pressure, nurses with more innovation attempts usually accumulate richer practical experience, which is positively correlated with better professional judgment and higher overall competency (Yan et al., 2022).

There was a notable positive correlation between innovative behavior and knowledge sharing behavior (*r* = 0.268, *p* < 0.05), reflecting that higher innovative behavior co-occurs with greater knowledge exchange engagement among nurses. According to Cohen’s standard for correlation coefficient effect size, an r value between 0.10 and 0.29 represents a small effect size, and r ranging from 0.30 to 0.49 indicates a medium effect size. The correlation coefficient *r* = 0.268 in this study falls within the small-to-medium range, revealing a weak to moderate positive linkage between nurses’ innovative behavior and knowledge sharing. Consistent with the positive linear association between knowledge sharing and nurses’ innovative behavior validated in this study, Zhou et al. (2026) also reported a strong positive correlation between the two variables based on aal nurse sample. Innovation frequently involves considering the present situation and merging information from various fields, and often requires a deep dive into personal expertise and a wide-ranging synthesis of team insights. Innovative nurses are inclined to actively search for new knowledge and technologies, gathering resources and inspiration for innovation by engaging in knowledge sharing (Zhu et al., 2025). Furthermore, The effectiveness of innovative practices frequently depends on collective wisdom, knowledge sharing was positively correlated with higher innovation levels, possibly through enabling the exchange of personal insights and feedback. Additionally, Organizations that prioritize innovation often support open communication and collaborative efforts, thereby reinforcing the symbiotic connection between innovation and sharing.

Consist with Leng et al. (2026), there was a significant positive correlation between knowledge sharing behavior and nursing competency (*r* = 0.365, *p* < 0.01). Per Cohen’s thresholds, *r* = 0.365 represents a medium effect size, showing a moderate linkage between the two indicators, indicating that knowledge sharing co-varies with nurses’ professional capabilities, and this connection exists separately from individual learning activities. Through structured communication, experience transfer, and collective reflection, nurses not only rapidly acquire practical experience and expertise while shortening their learning curve, but also internalize implicit clinical decision-making frameworks, interpersonal collaboration models, and professional values through interaction. This is positively correlated with higher overall competency scores (Zhu et al., 2025). These results emphasize the importance of encouraging knowledge-sharing habits to create teams focused on learning and enhance the exchange of knowledge among nursing teams.

### 4.3 The moderating role of knowledge sharing behavior between innovative behavior and nursing competency

The research utilized structural equation modeling to explore how knowledge sharing behavior moderates the link between clinical nurses’ innovative actions and their nursing skills. Consistent with the theoretical expectations raised in the Introduction, this moderating pattern can be interpreted from organizational learning theory and social exchange theory. From the perspective of organizational learning theory, team knowledge sharing integrates scattered innovative experience into collective clinical wisdom, forming a complete learning loop of “proposing ideas – exchanging experience – optimizing practice.” This explains why innovative behavior shows a stronger predictive linkage with nursing competency under high knowledge-sharing conditions. From the lens of social exchange theory, regular knowledge exchange builds mutual trust, peer feedback and emotional support among nurses. Such reciprocal interactions reduce barriers to implementing innovation, so innovation is barely associated with competency when knowledge sharing is scarce. The findings suggest that knowledge sharing behavior directly and positively influences nursing competency and significantly boosts the positive effects of innovative behavior on nursing competency. This suggests that in the professional field of nursing work, which is highly dependent on collaboration and knowledge integration, whether the association between innovative behavior and nursing competency is moderated by the level of knowledge sharing within the team to a certain extent (Liu et al., 2024). According to path analysis, the combination of innovative behavior and knowledge sharing behavior had a notable positive effect on nursing competency (*β* = 0.219, *p* = 0.031). Innovative behavior (*β* = 0.229) and knowledge sharing behavior (*β* = 0.224) each independently and directly predict nursing competency, highlighting their combined impact on competency enhancement. Subsequent elementary slope analysis demonstrated that in groups with elevated knowledge sharing behavior scores, the predictive effect of innovative behavior on nursing competency was more pronounced (*β* = 0.448, *p* < 0.001), significantly exceeding that in the low knowledge sharing group (*β* = 0.011, *p* = 0.909). The subgroup differentiation of simple slope results further supports the dual theoretical reasoning above and forms a closed logical chain with the hypotheses proposed in the Introduction. This suggests that a comprehensive knowledge-sharing environment can increase the beneficial impact of innovative behavior on nursing competence.

From a mechanistic viewpoint, the act of sharing knowledge probably moderates the predictive correlation of innovative behavior on nursing skills through various channels. Initially, higher knowledge sharing correlates with richer collective knowledge and information resources for innovation. Through written summaries, experience sharing, and community practice, individual and collective knowledge is integrated and circulated. This may assist nurses to make decisions based on more comprehensive information during the innovation process, potentially reducing trial-and-error costs while improving the effectiveness and applicability of innovative solutions (Lavoie, 2025). Secondly, the knowledge-sharing process is accompanied by social support and timely feedback. High levels of organizational communication and personal interaction provide innovators with emotional support, professional technical advice, and resource connections. This was associated with fewer obstacles during innovation implementation and more consistent innovative behaviors as reported by participants. Moreover, an environment that encourages knowledge sharing promotes the creation of learning teams and psychologically secure settings. In teams that encourage sharing and tolerate exploration, nurses are more willing to take risks associated with innovation and see challenges as opportunities for growth. This was associated with sustained innovative behavior and higher professional competency (Liu et al., 2024).

These results hold important consequences for the development of talent and nursing management. Nursing involves both standardized procedures and complex situations, which makes it challenging to enhance quality solely through skill training or institutional limitations. The proactive agency of nurses in improving practice is reflected in their innovative behavior, while sharing knowledge may play a vital role in transforming personal experiences into communal wisdom (Hanners et al., 2023). The absence of efficient knowledge sharing methods often keeps innovative outcomes at the individual level, preventing their dissemination and team-wide empowerment. Therefore, nursing managers should emphasize building a culture of open, collaborative, and institutionalized knowledge sharing to drive nurses’ innovation. This can be achieved by establishing structured case discussion mechanisms, building and maintaining departmental knowledge databases, creating platforms for sharing innovative experiences, and fostering interdisciplinary and multi-professional academic and practical interactions. Furthermore, for nurse groups exhibiting deficient knowledge sharing behaviors, it is imperative to identify and address barriers, such as time constraints, absence of incentives, and psychological concerns, through institutional support, leadership modeling, and team-building initiatives.

## 5 Conclusion

This study identifies that knowledge sharing behavior positively moderates the correlational link between clinical nurses’ innovative behavior and nursing competency. Innovative behavior was identified as a crucial correlate of nursing competency, particularly in environments rich in knowledge sharing. The underlying mechanisms may involve knowledge sharing providing an informational foundation, social support, and a psychologically safe environment for innovation, which may facilitate the conversion of tacit knowledge and team learning.

Nursing managers need to develop a structured framework for knowledge sharing to support the organizational process that links innovative behavior with competency. This study has certain limitations, including the adoption of convenience sampling without random assignment, which may reduce sample representativeness and restrict the generalizability of research conclusions, the cross-sectional design cannot establish causal relationships, only correlational and moderating associations can be observed, relatively concentrated sample source, and the need to expand the measurement dimensions of knowledge sharing behavior to include emerging forms of knowledge sharing in digital and intelligent environments. In addition, only the moderating model was tested; mediation/moderated-mediation comparisons could not be conducted due to anonymized ethical data and fixed questionnaire design, as knowledge sharing theoretically serves as a boundary moderator rather than a mediator. Future investigations could utilize longitudinal designs, expand the diversity of samples, verify the dynamic moderating mechanisms between innovative actions and nursing proficiency, and further examine competing path models.

## Data Availability

The raw data supporting the conclusions of this article will be made available by the authors, without undue reservation.
